# Microarray Analysis Reveals Sepsis Is a Syndrome with Hyperactivity of TH17 Immunity, with Over-Presentation of the Treg Cell Cytokine TGF-β †

**DOI:** 10.3390/cimb47060435

**Published:** 2025-06-09

**Authors:** Yu-Ju Chen, Jang-Jih Lu, Chih-Pei Lin, Wan-Chung Hu

**Affiliations:** 1 Department of Laboratory Medicine, Taipei Tzu Chi Hospital, Buddhist Tzu Chi Medical Foundation, No. 289 Jianguo Road, Xindian District, New Taipei City 231, Taiwan; 2 Department of Clinical Pathology, Taipei Tzu Chi Hospital, Buddhist Tzu Chi Medical Foundation, No. 289 Jianguo Road, Xindian District, New Taipei City 231, Taiwan; 3 Department of Medical Research, Taipei Tzu Chi Hospital, Buddhist Tzu Chi Medical Foundation, No. 289 Jianguo Road, Xindian District, New Taipei City 231, Taiwan; 4 Department of Biotechnology, Ming Chuan University, Taoyuan City 333, Taiwan

**Keywords:** sepsis, Th17, innate immunity, adaptive immunity, Treg

## Abstract

Currently, there are two major theories regarding the pathogenesis of sepsis: hyperimmune and hypoimmune. The hyperimmune theory suggests that a cytokine storm causes the symptoms of sepsis. On the contrary, the hypoimmune theory suggests that immunosuppression causes the manifestations of sepsis. By conducting a microarray analysis on peripheral leukocytes from patients with sepsis, this study found that hyperactivity of TH17 immunity was noted in sepsis patients. Innate immunity-related genes are significantly upregulated, including *CD14*, *TLR1*,*2*,*4*,*5*,*8*, *HSP70*, *CEBP* proteins, AP1 (*JUNB* and *FOSL2*), *TGFB1*, *IL6*, *TGFA*, *CSF2* receptor, *TNFRSF1A*, S100A binding proteins, *CCR2*, *FPR2*, amyloid proteins, pentraxin, defensins, CLEC5A, whole complement machinery, *CPD*, *NCF*, *MMP*, neutrophil elastase, caspases, IgG and IgA Fc receptors (*CD64*, *CD32*), *ALOX5*, *PTGS*, *LTB4R*, *LTA4H*, and *ICAM1*. The majority of adaptive immunity genes were downregulated, including MHC-related genes, TCR genes, granzymes/perforin, *CD40*, *CD8*, *CD3*, TCR signaling, BCR signaling, T and B cell-specific transcription factors, NK killer receptors, and TH17 helper-specific transcription factors (*STAT3*, *RORA*, and *REL*), as well as Treg-related genes, including *TGFB1*, *IL15*, *STAT5B*, *SMAD2/4*, *CD36*, and thrombospondin. The findings of this study show that Th17 with Treg over-presentation play an important role in the pathophysiology of sepsis.

## 1. Introduction

Despite the development of antibiotics, the mortality rate associated with sepsis remains high. However, the exact pathophysiology of sepsis remains unclear. Currently, there are two dominant theories explaining the etiology of sepsis: hyperimmune and hypoimmune theories. However, these two theories are mutually contradictory. The hyperimmune theory, proposed by Dr. Lewis Thomas, states that the hyperactivation of proinflammatory cytokines, known as the cytokine storm, is the actual cause of sepsis symptoms. These uncontrolled cytokines cause severe damage to several organs, leading to multiple organ failure. Based on this theory, clinical trials of therapeutic strategies, such as antibody-neutralizing TNF-α or anti-interleukin 1 therapies, were conducted in patients with sepsis. However, these antibodies do not improve the survival rate of patients with sepsis [1,2,3,4]. Furthermore, in a clinical trial, anti-TNF-α increased the mortality rate of patients with sepsis [2]. This created doubts regarding the hyperimmune theory. These failed clinical trials pointed out that hyperimmune is not the sole factor in the pathophysiology of sepsis. Thus, the hypoimmune theory emerged. Based on the observation that immunosuppressed patients are prone to developing sepsis, a hypoimmune status was suggested as the etiology of sepsis. However, the hypoimmune theory cannot satisfactorily explain the proinflammatory cytokine storm observed in sepsis. Both the hyperimmune and hypoimmune theories are supported by clinical and experimental evidence. This points out the complexity of the pathogenesis of sepsis. However, we still need to find an alternate theory to help explain the pathologic events of sepsis. In a previous study, we proposed an entire framework for host immunological pathways, including eradicable and tolerable immune reactions [5,6,7,8,9,10,11,12]. Here, we used a microarray study of whole blood from patients with sepsis to put forth a new theory that proposes that sepsis is a syndrome of Th17 immunity with the over-presentation of proinflammatory cytokines as well as Treg cells. Th17 immunity is a tolerable immune reaction against extracellular microorganisms, including extracellular bacteria [5]. It is triggered by both immune-stimulant interleukin-6 and immune-suppressor TGF-β. If Th17 is the key immune pathway related to sepsis, then we can combine both the hyperimmune and hypoimmune theories in the pathogenesis of sepsis [13,14]. The new theory addresses the existing controversy regarding the etiology of sepsis.

## 2. Materials and Methods

### 2.1. Microarray Dataset

In a study by Dr. J. A. Howrylak, total RNA was collected from the whole blood of patients with sepsis and sepsis-induced ARDS [15]. Patients were recruited from the Medical Intensive Care Unit of the University of Pittsburgh Medical Center between February 2005 and June 2007. Patients admitted to the Medical Intensive Care Unit for less than 48 h who were intubated and received mechanical ventilation were eligible for the study. The patients were classified as having sepsis if they met the criteria for sepsis, as defined by the Society of Critical Care Medicine Consensus statement [16]. This study attempted to identify the molecular signature of ARDS in comparison to patients with sepsis. This dataset is available from the Gene Expression Omnibus (GEO) www.ncbi.nlm.nih.gov/geo (accessed on 12 May 2025; accession number GSE 10474). Samples of patients with sepsis from this dataset were used for further microarray analysis. There was a total of 21 patients with a mortality rate of 35%. This further analytic study included patients with sepsis only and sepsis with ARDS.

The second dataset was obtained from GSE20189 of the Gene Expression Omnibus. This dataset was collected by Dr. Melissa Rotunno of the Cancer Prevention Research in 2011 [17]. The molecular signature of the early stages of lung adenocarcinoma was studied using microarray analysis. From this dataset, the data from whole blood RNA of the healthy controls (n = 21) was compared with those of patients with sepsis obtained from the previously mentioned dataset. In this study, further analysis was performed to analyze the peripheral leukocyte gene expression profiles of patients with sepsis compared with those of healthy controls. Although the above two datasets were from different studies, they both used Affymetrix HG-U133A 2.0 genechips under good quality control. In addition, the usage of RMAExpress software (version 1.2.0) can eliminate the possible batch effects for RMA normalization across these datasets.

### 2.2. Statistical Analysis

Affymetrix HG-U133A 2.0 genechip was used for both samples. RMAExpress software (UC Berkeley, Board Institute) was used for normalization and to rule out outliers in the dataset. Samples that exceeded the 99% line in the RLE-NUSE T2 plot were removed as potential outliers.

GeneSpring XI software (version 12) was used to analyze the significantly expressed genes between ARDS and healthy control leukocytes. Significance was set at *p* < 0.05. A fold-change cutoff of >2.0 was considered the cutoff for differential expression. The Benjamini–Hochberg-corrected false discovery rate (default value 0.05) was used during the analysis. These cutoff values are standardly used in microarray studies. In total, a list of 3277 genes was generated from the HGU133A2.0 chip, with 18,400 transcripts, including 14,500 well-characterized human genes.

### 2.3. RT-PCR Confirmation

Dr. J. A. Howrylak performed real-time PCR for the selected transcripts (cip1 and kip2) using TaqMan Gene Expression Assays (Applied Biosystems, Foster City, CA, USA). For the second dataset, Dr. Melissa Rotunno performed qRT-PCR to validate the microarray results. The RNA quantity and quality were determined using an RNA 600 LabChip-Aligent 2100 Bioanalyzer (Agilent, Santa Clara, CA, USA). RNA purification was performed using reagents from Qiagen (Hilden, Germany). All real-time PCRs were conducted using an ABI Prism 7000 Sequence Detection System (Waltham, MA, USA) with the designed primers and probes for the target genes, with *GAPDH* serving as the internal control gene. This confirmed that the microarray results were convincing compared to the RT-PCR results.

## 3. Results

### 3.1. RMA Analysis of Whole Blood

RMA was performed on RNA samples from the whole blood of the healthy controls from the lung adenocarcinoma dataset and from the whole blood of the patients from the sepsis dataset. A raw boxplot, a NUSE plot, an RLE value plot, an RLE-NUSE multiplot, and an RLE-NUSE T2 plot were generated. Samples were then included or excluded based on these graphs. Owing to a strong deviation in the T2 plot, sample GSM506435 from the lung adenocarcinoma dataset was excluded from further analyses. Similarly, GSM265024 and GSM265030 from the sepsis dataset were also excluded.

### 3.2. Toll-like Signaling and Heat Shock Protein Expression in Patients with Sepsis

Microarray analysis revealed that Toll-like receptors (TLRs) 1, 2, 4, 5, and 8 were upregulated in the patients with sepsis (Table 1). CD14 and downstream signaling molecules, such as *IRAK4* and *TAB2*, were also upregulated. TLRs 1, 2, 4, 5, and 8 mediate antibacterial immune responses, thereby triggering TH17-like proinflammatory cytokines, such as IL-6. However, the negative TLR regulator *IRAK3* was 21-fold upregulated. Thus, TLR 1, 2, 4, 5, and 8 signaling may not successfully trigger proinflammatory cytokines. Other pathways, such as CD14, may act as important alternative pathways that trigger IL-6 and other TH17-like cytokines. Other pattern recognition receptors, such as formyl peptide receptors (FPRs), which recognize specific bacterial antigens to trigger innate immunity, were also differentially expressed. *FPR1* was 7.6-fold downregulated, whereas *FPR2* was 4.7-fold upregulated.

### 3.3. Antigen Processing and Antigen Presentation Genes in Sepsis

All MHC-related genes were downregulated in the leukocytes of the patients with sepsis (Table 2). These downregulated genes included *HLA-DPB*, *HLA-DQA*, *HLA-DRB*, HLA-DOB, HLA-DRA, Tapasin, MHC-related transcripts, *HLA-B*, and *HLA-DPA*. Among these, *HLA-B* was more than 11-fold downregulated. MHC genes are key in antigen presentation to trigger adaptive immune reactions, such as B or T cell activation. As all MHC-related genes were downregulated, antigen presentation during sepsis is likely to be impaired. This agrees with previous observations [18].

### 3.4. TH17-like Innate Immune Transcription Factors in Sepsis

Many immune-related transcription factors were differentially regulated in the patients with sepsis (Table 3). First, several innate immunity-related transcription factors were upregulated in the patients with sepsis. These included AP1 (*JUNB* and *FOSL2*), *NFIL3*, *ARNT*, and *CEBP* (*CEBPA*, *CEBPG*, and *CEBPD*). The aryl hydrocarbon receptor nuclear translocator (ARNT) plays an important role in activating TH17-like innate immunity. The CEBP family of genes is related to the activity of myeloid cells and granulocytes. CEBP genes are also involved in the activation of acute response proteins. In addition, NFKBIA, an inhibitor of NF-κB, is downregulated in sepsis. This indicated that the activity of NF-κB, a key innate immunity mediator, was upregulated in the patients with sepsis. It is worth noting that two important transcription factors, i.e., High Mobility Group Box (*HMGB*) and Hypoxia-inducible factor alpha (*HIFA1*), are also upregulated during sepsis. HMGB, a vital innate immunity mediator, was upregulated by more than nine-fold.

STAT1, a key transcription factor for TH1 and THαβ immunity, is downregulated in sepsis. Additionally, TBX21 (T-bet), a key driver of the TH1 immune response, is also downregulated, whereas MafB, which can suppress IFNαβ in THαβ immunity, is upregulated [19]. Other TH2-related key transcription factors, such as GATA3 and C-MAF, are also downregulated [20]. This means that TH1, TH2, and THαβ are downregulated in sepsis. Interestingly, key TH17-related transcription factors, including REL, STAT3, and RORA, are also downregulated [21]. Moreover, SOCS3, a negative regulator of the central TH17 transcription factor STAT3, is upregulated, indicating that TH17 helper cells cannot be successfully triggered. On the other hand, Treg and TGFβ signaling molecules, including STAT5B, IL-15, SMAD2, and SMAD4, are upregulated [22,23]. TH17 and Treg-associated ARNT are also upregulated in sepsis [24]. Thus, Tregs are likely to be activated during sepsis. This is consistent with previous observations that Tregs are upregulated during sepsis.

The downregulated genes include B cell stimulatory transcription factor (*PAX5*), BCR signaling (*FYN* and *LYN*), and PI3K signaling (*PIK3CB*, *PIK3IP1*, *PIK3CG*, and *PIK3R1*) [25,26,27]. *PTEN*, a negative regulator of PI3K signaling, was 4.6-fold upregulated. BCL6 is a key transcription factor of follicular helper T cells in IgM-producing B cells. IBTK can inhibit B cell differentiation and activation. PI3K signaling is a downstream stimulatory pathway of B cell activation. Thus, BCR signaling appears to be suppressed during sepsis.

### 3.5. TH17-like and Treg-Related Cytokines Are Upregulated During Sepsis

Many TH17-like and Treg-related cytokines were upregulated in the patients with sepsis (Table 4). The whole TGFβ activation machinery was upregulated, including *THBS1*, *CD36*, and *TGFB1* itself. *TGFA* and *IL15* were also upregulated. *IL6* was also upregulated in sepsis. Thus, both key TH17-driven cytokines, i.e., TGFβ and IL-6, are activated in patients with sepsis. However, the full activation of TH17 helper cells also requires TCR signaling. IL32, a TH1-related macrophage differentiation factor [28], was also downregulated. Among TH22 mediators, *IL1A* was downregulated, whereas *IL1RN* (an IL1 receptor antagonist) was upregulated. This indicated that TH22 is not activated during sepsis.

Cytokine receptors were differentially regulated during sepsis (Table 5). Contrary to what was observed with cytokines, cytokine receptors in a certain immunological pathway were downregulated. Thus, TH17-like immunity was activated, whereas *TGFBR3*, *IL6R*, and *IL17RA* were downregulated. *TGFBR3* was downregulated by more than 11-fold, and *IL6R* was downregulated by more than 16-fold. Tregs were also activated, whereas *TGFBR3*, *IL2RB*, and *IL7R* were downregulated. TH1-related cytokine receptors *IFNGR1* and *IFNGR2* were upregulated. The TH2 cytokine receptor *IL4R* was also upregulated. With regard to TH-αβ immunity, *IFNAR1* was upregulated, whereas *IFNAR2* was downregulated. TH22 cytokine receptors *IL1R1* and *IL1R2* were upregulated. These findings indicate that TH1, TH2, TH-αβ, and TH22 are not activated during sepsis.

### 3.6. Upregulation of Th17-like Innate Immunity-Related Effector Molecules During Sepsis

Several acute response proteins were upregulated (Table 6). These acute phase proteins are upregulated by IL6 and CEBP. These genes included amyloid proteins (*APP* and *APLP2*), pentraxin (*PTX3*), transferrin receptor (*TFRC*), CLEC (*CLEC5A* and *CLEC1B*), and defensins (*DEFA1*, *DEFA1B*, *DEFA3*, and *DEFA4*). These are innate immunity effector proteins that attack bacterial antigens in a nonspecific manner. Defensin A4 (*DEFA4*) was upregulated by more than six-fold.

The entire complement machinery, an important effector component of innate immunity, was upregulated (Table 7). These included *CD59*, *CD55*, *C1QB*, *ITGAM*, *CR1*, *CD46*, *C3AR1*, *ITGAX*, *C1QA*, *C1RL*, *C5AR1*, and *CD97*. Therefore, complement molecules are activated during sepsis. These complement molecules attack bacterial cell walls and membranes, causing damage. However, complements may also have harmful effects on the host.

PMN matrix metallopeptidases (MMPs) and elastases were upregulated (Table 8). These enzymes can digest bacterial antigens as well as the extracellular matrix. These genes included *MMP8*, *MMP9*, *MMP25*, and *ELANE* (elastase). In addition, tissue inhibitors of MMP and TIMP2 and serum inhibitors of elastase or proteinase, *SERPINA1*, *SERPINB1*, and *SERPINB2*, were upregulated. This indicated that PMN proteinases are dysregulated during sepsis. It is worth noting that *MMP8* was 32-fold upregulated and *MMP9* was 10-fold upregulated.

### 3.7. Dysregulation of Coagulation-, Glycolysis-, Acidosis-, and Vasodilation-Related Genes in Sepsis

Many coagulation-related genes were dysregulated during sepsis (Table 9). Disseminated intracellular coagulopathy (DIC) is a common manifestation of human sepsis. The upregulated coagulation-related genes included *F13A1*, *F5*, *F8*, *GP1BB*, *PROS1*, *PLAUR*, *MCFD2*, *TFPI*, *F2RL1*, *ITGA2B*, *PDGFC*, *ITGB3*, and *THBD*. This indicates that both coagulation factors and coagulation inhibitors are dysregulated during sepsis.

All glycolytic pathway enzyme genes were upregulated during sepsis (Table 10). These included lactate dehydrogenase A, phosphoglycerate kinase 1, pyruvate kinase, 6-phosphofructo-2-kinase/fructose-2,6-biphosphatase 3, hexokinase 2, glycogen phosphorylase, 2,3-bisphosphoglycerate mutase, hexokinase 3, glucose-6-phosphate isomerase, 6-phosphofructo-2-kinase/fructose-2,6-biphosphatase 2, glyceraldehyde-3-phosphate dehydrogenase, enolase 1, and phosphoglycerate kinase 1. In addition, pyruvate dehydrogenase kinase, which prevents the conversion of pyruvate to acetyl-CoA, was upregulated. Pyruvate dehydrogenase phosphatase, which facilitates the conversion of pyruvate to acetyl-CoA to enter the aerobic citric acid cycle, was downregulated in sepsis. Therefore, the retention of pyruvate can facilitate lactate formation in the anaerobic pathway during sepsis.

Concurrently, H^+^-ATPases were upregulated in sepsis (Table 11). In a previous article, we determined the coupling between glycolytic enzymes and H^+^-ATPases during falciparum malarial infection. Consistently, this study also revealed upregulated expression of H^+^-ATPases, including *ATP6V0B*, *ATP6V0E1*, *ATP6AP2*, *ATP6V1C1*, *TCIRG1*, *ATP6V1D*, *ATP11B*, and *ATP11A*. In addition, carbonic anhydrases IV and II (*CA4* and *CA2*), which produce H_2_CO_3_, were upregulated in sepsis. This can help explain acidosis during sepsis.

### 3.8. Failure of T-Lymphocyte Adaptive Immunity During Sepsis

Lymphocytes play important roles in adaptive immunity. In sepsis, the major lymphocyte populations, including T and B cells, were downregulated. Thus, adaptive lymphocyte immunity is not induced during sepsis. This is very important in the pathogenesis of sepsis.

Many T cell-related genes were also downregulated (Table 12). These downregulated genes included those associated with TCR (*TRAC*, *TARP*, *TRBC1/C2*, *TRD@*, *TRGC2*, and *TRDV3*), CD costimulatory molecules (*CD3E*, *CD8A*, *CD3G*, *LY9*, *CD3D*, and *CD2*), T cell-specific transcription factors (*IKZF1*, *TCF7*, *NFAT5*, *NFATC3*, *TCF7L2*, *NFATC2IP*, *TBX21*, *ID2*, and *ID2B*), granzyme/perforin (*GZMA*, *GNLY*, *GZMK*, *GZMB*, *GZMH*, and *PRF1*), and TCR downstream signaling (*ZAP70* and *LCK*) [29]. Thus, the entire T cell activation machinery is suppressed. Both CD4 helper T cells and CD8^+^ cytotoxic T cells are inactivated and downregulated in patients with sepsis.

### 3.9. Results of Ingenuity Pathway Analysis

**Figure 1:**
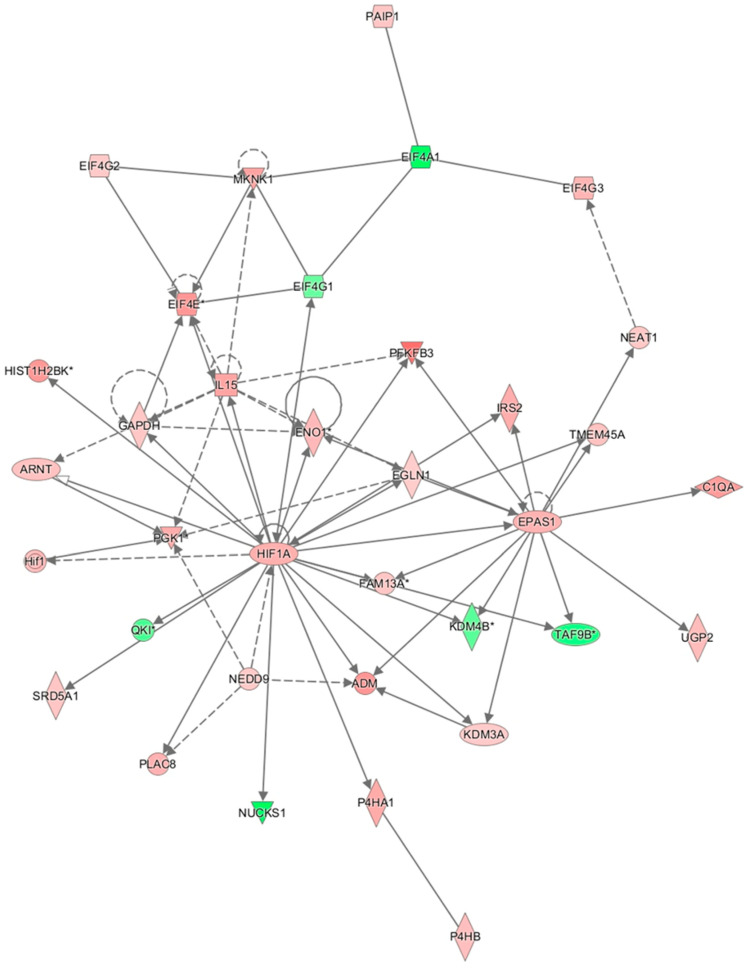
HIF-centered network in sepsis via ingenuity pathway analysis. Solid line means direct regulation and dot line means indirect regulation. * means this gene has high fold change value.

**Figure 2:**
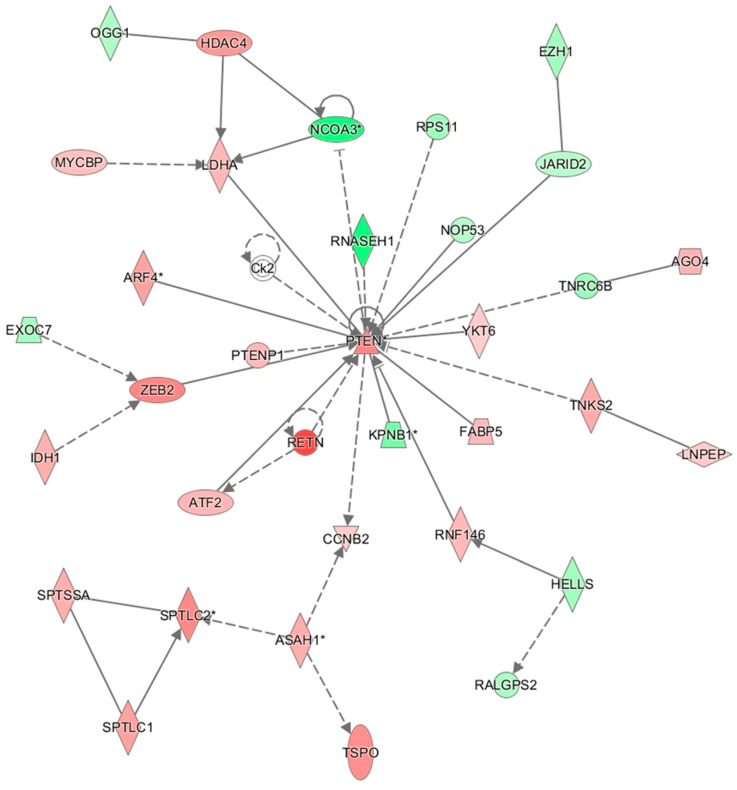
PTEN-centered network for sepsis via ingenuity pathway analysis. Solid line means direct regulation and dot line means indirect regulation. * means this gene has high fold change value.

**Figure 3:**
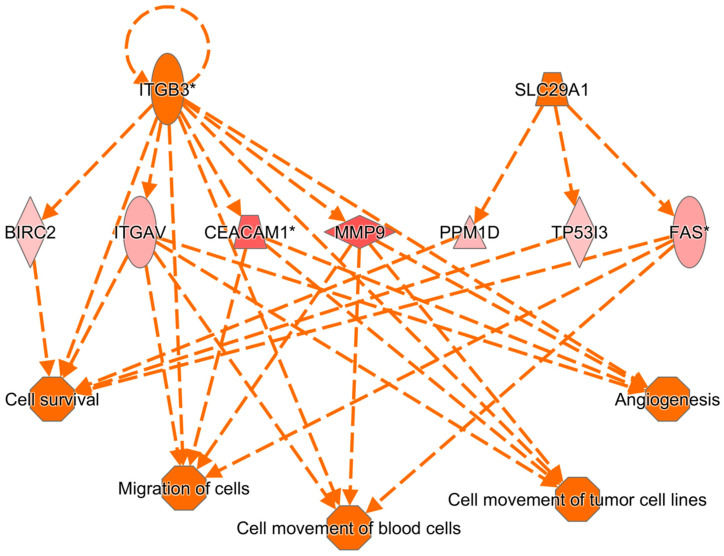
ITGB3- and MMP9-dominant regulatory pathways in sepsis via ingenuity pathway analysis. Arrow means up-regulation and * sign means high fold change value.

**Figure 4:**
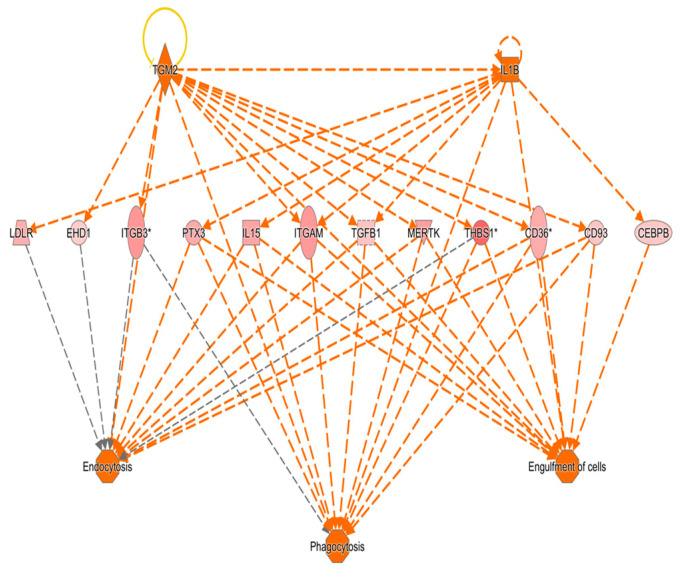
IL1B-, TGFB-, and TGM2-dominant regulatory pathways in sepsis via ingenuity pathway analysis. Arrow means up-regulation, yellow circle means auto-suppression, and * means high fold change value.

**Figure 5:**
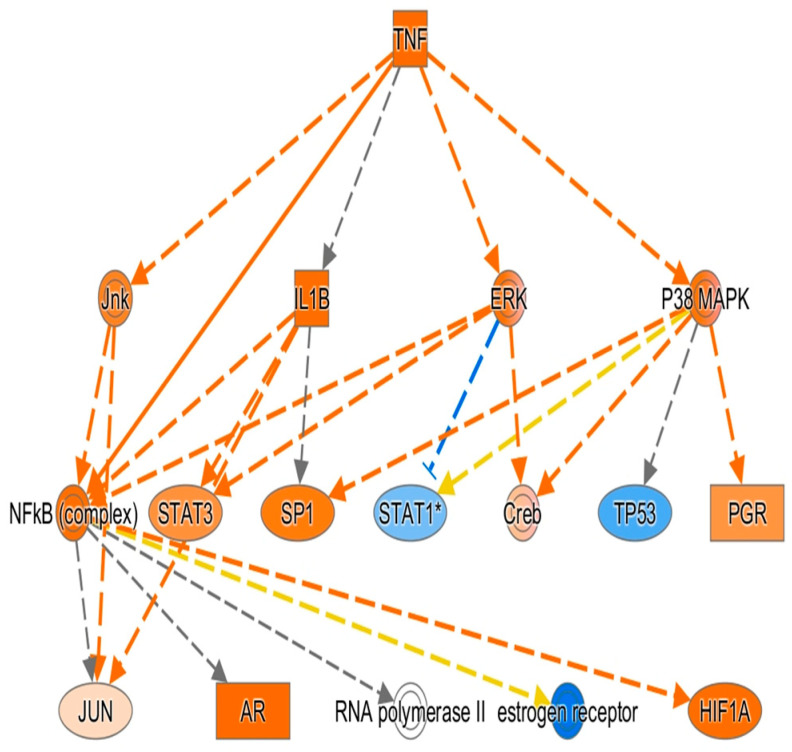
TNF is the key upstream mediator during sepsis, as identified via ingenuity pathway analysis. Orange dotted line means activation, blue dotted line means inhibition, yellow dotted line means inconsistent with expression, and grey line means unclassified interaction. * sign means high value in fold change.

In the network analysis, the most over-represented network was the HIF1A-centered network, and the second-most over-represented network was the PTEN-centered network (Figure 1 and Figure 2). Sepsis is related to tissue hypoxia, and PTEN is related to immunosuppression. In Figure 3 and Figure 4, the top regulator effector networks are shown, including ITGB3, IL1B, and TGFB. ITGB3 and IL1B play important roles in innate immunity. TGFB also plays an important role in immunosuppression. TGFB is located at the center of the regulator effector network. Therefore, both innate immunity and immunosuppression are important in the pathogenesis of sepsis. As shown in Figure 5, the upstream regulator identified in sepsis was TNF, which also suggests that innate immunity is key to the pathophysiology of sepsis.

## 4. Discussion

Despite current antibiotic treatments, sepsis has a high mortality rate. However, its pathophysiology remains unclear [30,31]. The dominant theory regarding the mechanism of sepsis is the hyperimmune theory [32]. Hyperimmunity with a cytokine storm was observed in sepsis by Dr. Lewis Thomas [33]. He suggested that the symptoms and signs of sepsis were due to the overactivity of proinflammatory cytokines. This theory has been widely accepted. Based on the hyperimmune theory, many therapeutic strategies have been developed. The most well-known approach is the use of anti-TNF agents in clinical trials of sepsis. Because the proinflammatory cytokine TNFα is upregulated in sepsis, the use of anti-TNF agents should have contributed to the control of sepsis. However, the opposite results were observed. The use of anti-TNF agents has been shown to increase the mortality rate associated with sepsis [2,34,35]. This casts doubts over the sepsis hyperimmune theory.

Other theories on sepsis pathophysiology have also emerged, notably the hypoimmune theory. Because immunocompromised patients are prone to developing sepsis, hypoimmunity may be a cause of sepsis [36]. In addition, massive effector lymphocyte apoptosis, depletion of dendritic cells, and elevated Tregs have been observed during sepsis [37,38,39,40,41]. In previous reports, the downregulation of costimulatory molecules and MHC was noted in patients with sepsis [42]. In addition, B cells play important roles in the recovery from sepsis [43]. However, this hypothesis has not been accepted by most scientists because it does not explain the observed cytokine storm during sepsis. Thus, both theories are supported by some evidence and yet are only partially correct.

Thus, a third theory was proposed—the sequence theory. According to this theory, hyperimmunity occurs first during sepsis, followed by hypoimmunity. This theory attempts to integrate the two theories. However, it is unclear why such a sequential immune response occurs, and there is no existing immunological mechanism to explain this sequential effect. Why does hyperimmunity occur first, and why does hyperimmunity change into hypoimmunity? Furthermore, how do immunodeficient patients easily develop sepsis, and how do these patients easily develop a hyperimmune response initially? The current sepsis theory cannot explain this phenomenon.

In this study, we used microarray analysis to demonstrate that sepsis is characterized by hyperactivity of innate immunity and hypoactivity of adaptive immunity. This can explain the coexistence of hyperimmunity and hypoimmunity. Hypoactivity of adaptive immunity explains why immunocompromised patients tend to easily develop sepsis, while hyperactivity of innate immunity explains why a proinflammatory cytokine storm is observed in sepsis. Adaptive immune dysfunction, with a lack of T helper cells, is the key to the pathogenesis of sepsis. TH22 cells mediate eradicable immunity against extracellular bacteria, whereas TH17 cells mediate tolerable immunity against extracellular bacteria. Thus, blocking TH22-related cytokines, such as TNFα, can inhibit the further generation of TH22 helper cells to initiate adaptive immunity to combat or eradicate extracellular bacteria. This can explain why TNF blockade increases the mortality rate of patients with sepsis.

Previous studies have shown that TH22 immunity can successfully combat sepsis [44,45,46]. TH17 immunity comprises both IL-17-dominant proinflammatory cytokines and the TGF-β-dominant regulatory T cells. Thus, sepsis cannot activate host-eradicable immunity to completely kill the bacteria. On the other hand, sepsis triggers host-tolerable immunity with hyperimmune cytokine storms and hypoimmune TGF-β. The upregulation of TGF-β can cause multiorgan failure by promoting tissue fibrosis [11]. This explains why sepsis is usually associated with multiorgan failure. It is hypothesized that the TH17/Treg ratio determines the severity of sepsis [13,47]. However, this is unlikely because the TH17 immune response itself already includes a Treg cell component. TH17 is initiated by TGF-β plus IL-6 or other proinflammatory cytokines. It is possible that these pathogenic bacteria trigger the host TH17 rather than TH22 immunity. Importantly, eradicable TH22 immunity needs to be successfully induced to completely destroy extracellular bacteria.

This microarray study provided evidence to support our theory. Whole blood samples from patients with sepsis reflected leukocyte expression patterns. Innate immunity-related genes were significantly upregulated. These genes included *CD14*, *TLR1*, *2*, *4*, *5*, and *8*, *HSP70*, *CEBP*, AP1 (*JUNB* and *FOSL2*), *TGFB1*, *IL6*, *TGFA*, *CSF2* receptor, *FPR2*, amyloid proteins, pentraxin, defensins, *CLEC5A*, whole complement machinery, *CPD*, *NCF*, *MMP*, and neutrophil elastase. We also found that the majority of adaptive immunity genes were downregulated, including MHC-related genes, TCR genes, granzymes/perforin, CD40, CD8, CD3, TCR signaling, BCR signaling, T and B cell-specific transcription factors, and TH22 helper-specific transcription factors (STAT3, RORA, and REL). In addition, Treg-related genes were upregulated, including *TGFB*, *IL15*, *STAT5B*, *SMAD2/4*, *CD36*, and thrombospondin. Upregulation of regulatory cells during sepsis has also been reported previously. Upregulation of Treg-related genes can also suppress adaptive immunity during sepsis. These findings support the proposed theory of sepsis pathogenesis. This analysis confirmed a two-hit model of sepsis. The first hit triggers over-activated innate immunity. The second hit suppresses MHC and T helper cells to upregulate immunosuppression by regulatory T cells. This study provides further insight into the pathophysiology of sepsis.

Sepsis is also associated with several complications, such as disseminated intravascular coagulation (DIC), hypotension/shock, and lactate acidosis [48]. In this microarray analysis, we found that many coagulation-related genes were upregulated during sepsis. including *F5*, *F8*, facto13, protein S, plasminogen receptor, *ITGA2B*, *ITGB3*, and thrombomodulin. This may help explain the mechanism of DIC during sepsis. The entire set of glycolytic enzymes, including *LDHA*, *PGK1*, *PKM2*, *PFKFB3*, *HK2*, *PYGL*, *BPGM*, *HK3*, *PDK3*, *GPI*, *PFKFB2*, *GAPDH*, and *ENO1*, were upregulated during sepsis. In addition, glycolytic enzyme-coupled H^+^-ATPase genes were upregulated. These findings explain the lactate acidosis observed during sepsis.

Bacteria have strategies to suppress host immunity for their survival, especially adaptive immunity [49]. In conclusion, after understanding the pathogenesis of sepsis, better preventive and therapeutic agents can be developed to control this disease. Impairment of adaptive immunity may be more important than the overactivation of innate immunity during sepsis. Medications to activate host adaptive immunity, such as T helper cells, can be potentially used to combat sepsis. In addition, therapeutic strategies can be developed to cope with sepsis-related complications, such as DIC and lactate acidosis. Hopefully, a day will come when sepsis will be overcome.

The limitation of this study is due to its methodology. First, the patient numbers are limited because of the original dataset collection from the Gene Expression Omnibus. A larger sample size in future work may help to confirm the analytic results of this study. Second, because this study is purely a microarray study, it can only reflect the functional genomic changes during sepsis. No DNA or protein data were used in this analysis. If we want to further confirm this research, we will need more methodological approaches, including proteomics research. This can be the future research direction of our research group. However, the RNA data from this microarray functional genomic analysis are still very informative. We believe this analysis can shed light on the pathogenesis of sepsis. However, because of the complexity of sepsis, we need more evaluations to further support this theory.

## Figures and Tables

**Table 1:** TLR.

Probe ID	*p*-Value	Arrow	Fold	Gene
201743_at	1.37 × 10^−4^	up	2.18	*CD14*
204924_at	1.45 × 10^−10^	up	3.38	*TLR2*
210166_at	9.16 × 10^−8^	up	2.40	*TLR5*
210176_at	0.001131	up	2.07	*TLR1*
213817_at	3.14 × 10^−13^	up	21.04	*IRAK3*
219618_at	1.89 × 10^−9^	up	2.69	*IRAK4*
220832_at	4.76 × 10^−9^	up	5.16	*TLR8*
221060_s_at	6.62 × 10^−7^	up	3.33	*TLR4*
212184_s_at	2.03 × 10^−5^	up	2.61	*TAB2*
221705_s_at	8.46 × 10^−10^	down	2.08	*SIKE1*
205118_at	1.05 × 10^−10^	down	7.61	*FPR1*
210772_at	2.06 × 10^−8^	up	4.78	*FPR2*

**Table 2:** MHC.

Probe ID	*p*-Value	Arrow	Fold	Gene
201137_s_at	5.80 × 10^−4^	down	2.09	*HLA-DPB1*
203290_at	2.56 × 10^−8^	down	5.19	*HLA-DQA1*
204670_x_at	6.77 × 10^−8^	down	2.85	*HLA-DRB1/B4*
205671_s_at	1.27 × 10^−4^	down	2.02	*HLA-DOB*
208306_x_at	1.53 × 10^−6^	down	2.44	*HLA-DRB1*
208894_at	8.06 × 10^−7^	down	2.76	*HLA-DRA*
209312_x_at	1.24 × 10^−6^	down	2.67	*HLA-DRB1/B4/B5*
209823_x_at	8.65 × 10^−4^	down	2.08	*HLA-DQB1*
210294_at	7.08 × 10^−10^	down	2.25	*TAPBP*
210528_at	1.28 × 10^−5^	down	2.57	*MR1*
211948_x_at	3.66 × 10^−28^	down	11.75	*BAT2L2*
211990_at	5.10 × 10^−6^	down	3.19	*HLA-DPA1*
212384_at	8.83 × 10^−15^	down	2.98	*HLABAT1*
212671_s_at	0.002545	down	2.27	*HLA-DQA1/A2*
214055_x_at	1.16 × 10^−24^	down	9.42	*BAT2L2*
215193_x_at	2.90 × 10^−6^	down	2.52	*HLA-DRB1/B3/B4*
221491_x_at	1.50 × 10^−6^	down	2.25	*HLA-DRB1/B3/B4/B5*

**Table 3:** Transcription factors.

Probe ID	*p*-Value	Arrow	Fold	Gene
201473_at	3.65 × 10^−9^	up	2.46	*JUNB*
201502_s_at	9.16 × 10^−7^	down	2.36	*NFKBIA*
202527_s_at	5.77 × 10^−9^	up	3.24	*SMAD4*
203077_s_at	4.90 × 10^−7^	up	2.37	*SMAD2*
203574_at	4.37 × 10^−10^	up	5.18	*NFIL3*
204039_at	4.62 × 10^−8^	up	2.06	*CEBPA*
204203_at	9.92 × 10^−7^	up	2.17	*CEBPG*
205841_at	1.02 × 10^−13^	up	4.66	*JAK2*
206036_s_at	8.46 × 10^−12^	down	4.75	*REL*
206359_at	3.22 × 10^−7^	up	2.09	*SOCS3*
206363_at	9.68 × 10^−6^	down	2.26	*MAF*
208991_at	1.49 × 10^−13^	down	3.22	*STAT3*
209604_s_at	2.74 × 10^−19^	down	6.55	*GATA3*
209969_s_at	2.12 × 10^−8^	down	4.75	*STAT1*
210479_s_at	5.21 × 10^−15^	down	7.85	*RORA*
212501_at	1.73 × 10^−7^	up	2.17	*CEBPB*
212550_at	7.19 × 10^−10^	up	2.52	*STAT5B*
213006_at	6.03 × 10^−10^	up	4.21	*CEBPD*
218221_at	1.49 × 10^−11^	up	2.35	*ARNT*
218559_s_at	9.49 × 10^−7^	up	3.35	*MAFB*
218880_at	5.34 × 10^−11^	up	3.75	*FOSL2*
208808_s_at	1.07 × 10^−11^	up	9.12	*HMGB*
200989_at	1.17 × 10^−6^	up	3.00	*HIF1A*
221969_at	9.96 × 10^−13^	down	4.20	*PAX5*
203140_at	3.09 × 10^−10^	up	3.69	*BCL6*
210105_s_at	9.37 × 10^−10^	down	3.32	*FYN*
210754_s_at	2.98 × 10^−10^	down	3.55	*LYN*
217620_s_at	4.31 × 10^−12^	down	2.79	*PIK3CB*
221756_at	5.52 × 10^−10^	down	2.62	*PIK3IP1*
204054_at	2.56 × 10^−10^	up	5.51	*PTEN*
206370_at	2.95 × 10^−9^	down	2.44	*PIK3CG*
212240_s_at	4.29 × 10^−13^	down	4.51	*PIK3R1*

**Table 4:** Cytokines.

Probe ID	*p*-Value	Arrow	Fold	Gene
201110_s_at	2.02 × 10^−9^	up	8.27	*THBS1*
203085_s_at	1.57 × 10^−8^	up	2.33	*TGFB1*
203828_s_at	7.88 × 10^−5^	down	2.13	*IL32*
205016_at	8.33 × 10^−10^	up	4.86	*TGFA*
205992_s_at	4.40 × 10^−6^	up	3.58	*IL15*
208114_s_at	7.75 × 10^−20^	down	5.85	*ISG20L2*
208200_at	3.06 × 10^−11^	down	4.80	*IL1A*
212195_at	3.90 × 10^−6^	up	2.67	*IL6ST*
209555_s_at	2.87 × 10^−5^	up	3.18	*CD36*
212657_s_at	2.96 × 10^−7^	up	2.31	*IL1RN*

**Table 5:** Cytokine receptors.

Probe ID	*p*-Value	Arrow	Fold	Gene
201642_at	1.42 × 10^−9^	up	2.32	*IFNGR2*
202948_at	5.77 × 10^−10^	up	6.46	*IL1R1*
203233_at	2.36 × 10^−10^	up	3.27	*IL4R*
204191_at	2.98 × 10^−7^	up	2.06	*IFNAR1*
204731_at	7.48 × 10^−21^	down	11.93	*TGFBR3*
204786_s_at	5.23 × 10^−19^	down	6.86	*IFNAR2*
205227_at	2.89 × 10^−5^	up	2.68	*IL1RAP*
205291_at	2.89 × 10^−8^	down	2.44	*IL2RB*
205707_at	1.73 × 10^−9^	down	2.41	*IL17RA*
205798_at	2.48 × 10^−24^	down	31.79	*IL7R*
205926_at	1.06 × 10^−9^	down	2.19	*IL27RA*
205945_at	1.49 × 10^−22^	down	16.69	*IL6R*
206618_at	4.70 × 10^−9^	up	12.92	*IL18R1*
207072_at	5.22 × 10^−8^	up	4.93	*IL18RAP*
211372_s_at	1.76 × 10^−8^	up	10.68	*IL1R2*
211676_s_at	6.66 × 10^−9^	up	4.61	*IFNGR1*
205159_at	1.17 × 10^−6^	up	2.51	*CSF2RB*
210340_s_at	4.36 × 10^−10^	up	2.30	*CSF2RA*

**Table 6:** Acute response proteins.

Probe ID	*p*-Value	Arrow	Fold	Gene
200602_at	3.75 × 10^−12^	up	4.38	*APP*
206157_at	8.31 × 10^−8^	up	3.27	*PTX3*
208691_at	0.001264	up	2.49	*TFRC*
208703_s_at	1.26 × 10^−7^	up	3.05	*APLP2*
219890_at	1.43 × 10^−12^	up	7.83	*CLEC5A*
220496_at	2.59 × 10^−7^	up	3.33	*CLEC1B*
205033_s_at	1.17 × 10^−5^	up	4.79	*DEFA1/A1B/A3*
207269_at	2.87 × 10^−5^	up	6.67	*DEFA4*
201943_s_at	7.91 × 10^−12^	up	6.94	*CPD*
204961_s_at	7.26 × 10^−8^	up	2.02	*NCF1/1B/1C*
207677_s_at	5.88 × 10^−10^	up	2.66	*NCF4*
214084_x_at	1.31 × 10^−8^	up	2.25	*NCF1C*

**Table 7:** Complements.

Probe ID	*p*-Value	Arrow	Fold	Gene
200985_s_at	4.85 × 10^−11^	up	6.59	*CD59*
201925_s_at	2.14 × 10^−7^	up	5.61	*CD55*
202953_at	7.01 × 10^−6^	up	2.53	*C1QB*
205786_s_at	5.02 × 10^−13^	up	4.05	*ITGAM*
206244_at	6.06 × 10^−12^	up	6.76	*CR1*
208783_s_at	0.004769	up	2.21	*CD46*
209906_at	7.48 × 10^−9^	up	4.34	*C3AR1*
210184_at	1.17 × 10^−6^	up	2.07	*ITGAX*
218232_at	1.52 × 10^−8^	up	3.97	*C1QA*
218983_at	7.83 × 10^−8^	up	2.64	*C1RL*
220088_at	9.13 × 10^−8^	up	2.49	*C5AR1*
202910_s_at	3.42 × 10^−7^	up	2.26	*CD97*

**Table 8:** MMPs.

Probe ID	*p*-Value	Arrow	Fold	Gene
203167_at	1.02 × 10^−13^	up	3.14	*TIMP2*
203936_s_at	2.89 × 10^−16^	up	10.59	*MMP9*
206871_at	1.04 × 10^−6^	up	5.39	*ELANE*
207329_at	3.41 × 10^−11^	up	32.06	*MMP8*
207890_s_at	1.30 × 10^−11^	up	3.11	*MMP25*
202833_s_at	2.83 × 10^−9^	up	2.78	*SERPINA1*
204614_at	5.64 × 10^−8^	up	3.07	*SERPINB2*
212268_at	8.64 × 10^−11^	up	5.64	*SERPINB1*

**Table 9:** Coagulation.

Probe ID	*p*-Value	Arrow	Fold	Gene
203305_at	2.16 × 10^−4^	up	2.18	*F13A1*
204714_s_at	1.87 × 10^−8^	up	3.93	*F5*
205756_s_at	2.79 × 10^−5^	up	2.08	*F8*
205871_at	7.54 × 10^−7^	down	3.12	*PLGLA/B1/B2*
206655_s_at	2.25 × 10^−8^	up	5.37	*GP1BB/SEPT5*
207808_s_at	6.30 × 10^−8^	up	2.88	*PROS1*
211924_s_at	5.53 × 10^−7^	up	2.33	*PLAUR*
212245_at	6.18 × 10^−7^	up	2.30	*MCFD2*
213258_at	1.07 × 10^−6^	up	2.35	*TFPI*
213506_at	0.002877	up	2.35	*F2RL1*
214415_at	1.30 × 10^−9^	down	5.54	*PLGLB1/B2*
216956_s_at	4.64 × 10^−5^	up	2.39	*ITGA2B*
218718_at	2.79 × 10^−10^	up	9.39	*PDGFC*
204627_s_at	1.30 × 10^−6^	up	4.18	*ITGB3*
203887_s_at	8.66 × 10^−9^	up	4.53	*THBD*

**Table 10:** Glycolysis.

Probe ID	*p*-Value	Arrow	Fold	Gene
200650_s_at	2.02 × 10^−9^	up	2.71	*LDHA*
200737_at	2.94 × 10^−11^	up	3.17	*PGK1*
201030_x_at	9.45 × 10^−5^	down	2.02	*LDHB*
201251_at	2.51 × 10^−10^	up	2.67	*PKM2*
202464_s_at	6.45 × 10^−9^	up	7.30	*PFKFB3*
202934_at	9.80 × 10^−14^	up	4.77	*HK2*
202990_at	2.15 × 10^−12^	up	4.20	*PYGL*
203502_at	1.24 × 10^−4^	up	3.67	*BPGM*
205936_s_at	5.17 × 10^−12^	up	4.99	*HK3*
206348_s_at	9.53 × 10^−11^	up	2.60	*PDK3*
208308_s_at	3.92 × 10^−9^	up	2.22	*GPI*
209992_at	3.99 × 10^−9^	up	11.77	*PFKFB2*
213453_x_at	2.13 × 10^−12^	up	2.18	*GAPDH*
217294_s_at	3.28 × 10^−6^	up	2.62	*ENO1*
218273_s_at	1.01 × 10^−7^	down	2.25	*PDP1*

**Table 11:** H^+^-ATPases.

Probe ID	*p*-Value	Arrow	Fold	Gene
200078_s_at	6.15 × 10^−13^	up	2.53	*ATP6V0B*
201171_at	4.49 × 10^−10^	up	2.48	*ATP6V0E1*
201443_s_at	5.84 × 10^−6^	up	2.33	*ATP6AP2*
201971_s_at	4.45 × 10^−13^	down	5.21	*ATP6V1A*
202872_at	1.95 × 10^−10^	up	6.18	*ATP6V1C1*
202874_s_at	6.99 × 10^−10^	up	5.72	*ATP6V1C1*
204158_s_at	5.14 × 10^−8^	up	2.07	*TCIRG1*
208898_at	2.66 × 10^−9^	up	2.41	*ATP6V1D*
213587_s_at	1.13 × 10^−8^	down	2.07	*ATP6V0E2*
206208_at	1.00 × 10^−11^	up	3.51	*CA4*
206209_s_at	4.18 × 10^−15^	up	7.98	*CA4*
209301_at	2.78 × 10^−6^	up	3.42	*CA2*
212536_at	4.38 × 10^−9^	up	4.21	*ATP11B*
213582_at	1.89 × 10^−8^	up	2.24	*ATP11A*

**Table 12:** T cell.

Probe ID	*p*-Value	Arrow	Fold	Gene
205255_x_at	3.09 × 10^−8^	down	2.96	*TCF7*
205456_at	5.31 × 10^−8^	down	2.88	*CD3E*
205488_at	1.01 × 10^−5^	down	2.87	*GZMA*
205495_s_at	5.33 × 10^−10^	down	4.38	*GNLY*
205758_at	1.20 × 10^−7^	down	3.26	*CD8A*
206666_at	1.84 × 10^−7^	down	3.45	*GZMK*
206804_at	1.10 × 10^−15^	down	5.12	*CD3G*
207460_at	3.78 × 10^−9^	down	2.50	*GZMM*
208003_s_at	5.52 × 10^−18^	down	12.04	*NFAT5*
209671_x_at	3.58 × 10^−8^	down	2.77	*TRAC*
209813_x_at	1.49 × 10^−9^	down	4.42	*TARP*
210164_at	8.91 × 10^−9^	down	3.76	*GZMB*
210321_at	8.94 × 10^−10^	down	5.80	*GZMH*
210370_s_at	1.34 × 10^−7^	down	2.48	*LY9*
210556_at	4.68 × 10^−8^	down	2.85	*NFATC3*
210972_x_at	1.78 × 10^−7^	down	2.88	*TRAC/J17/V20*
211796_s_at	6.35 × 10^−6^	down	2.93	*TRBC1/C2*
212759_s_at	3.98 × 10^−16^	down	3.93	*TCF7L2*
213193_x_at	2.53 × 10^−6^	down	2.92	*TRBC1*
213539_at	1.00 × 10^−8^	down	3.19	*CD3D*
214617_at	2.22 × 10^−6^	down	2.65	*PRF1*
216191_s_at	4.71 × 10^−7^	down	4.76	*TRDV3*
216920_s_at	2.28 × 10^−10^	down	5.34	*TARP/TRGC2*
217143_s_at	1.26 × 10^−8^	down	6.06	*TRD@*
217527_s_at	2.12 × 10^−13^	down	5.80	*NFATC2IP*
220684_at	7.39 × 10^−9^	down	2.08	*TBX21*
220704_at	2.15 × 10^−10^	down	5.69	*IKZF1*
214032_at	6.60 × 10^−8^	down	2.52	*ZAP70*
204891_s_at	4.58 × 10^−8^	down	3.31	*LCK*
205831_at	4.40 × 10^−10^	down	3.93	*CD2*
201565_s_at	8.13 × 10^−13^	down	4.17	*ID2*
213931_at	7.33 × 10^−8^	down	3.55	*ID2/2B*

## Data Availability

Data is contained within the article.
